# Defining Recovery After Gastrectomy: Insights from Longitudinally Collected Patient-Reported Outcomes

**DOI:** 10.1245/s10434-026-19733-8

**Published:** 2026-04-30

**Authors:** Maho Takayama, Koichi Tomita, Paula Marincola Smith, Shu-En Shen, Xin Shelley Wang, Xuemei Wang, Laura R. Prakash, Elsa Melissa Arvide, Connie To, Paul Mansfield, Brian D. Badgwell, Naruhiko Ikoma

**Affiliations:** 1 Department of Surgical Oncology, The University of Texas MD Anderson Cancer Center, Houston, TX USA; 2 Department of Colon & Rectal Surgery, The University of Texas MD Anderson Cancer Center, Houston, TX USA; 3 Department of Symptom Research, The University of Texas MD Anderson Cancer Center, Houston, TX USA; 4 Department of Epidemiology, The University of Texas MD Anderson Cancer Center, Houston, TX USA; 5 Department of Biostatistics, The University of Texas MD Anderson Cancer Center, Houston, TX USA

**Keywords:** MD Anderson Symptom Inventory Survey, Patient-reported outcomes, Gastrectomy, Postoperative symptom recovery, Robotic approaches

## Abstract

**Background:**

Longitudinal patient-reported outcomes (PROs) can capture symptom burden and functional recovery, but benchmarks across gastrectomy, including the impact of bodyweight loss on quality of life, remain unclear.

**Methods:**

We prospectively collected PRO data (October 2020–January 2025) from patients undergoing gastrectomy who completed the MD Anderson Symptom Inventory at eight time points, from preoperative through postoperative month (POM) 6. Symptom and interference composite scores were calculated as the mean of the top five symptoms and top three interference items identified at postoperative day (POD) 3. Recovery was defined as achieving mild scores (≤3) on both composites. Logistic regression identified factors associated with recovery at POM 1. Bodyweight changes from preoperative baseline were assessed at POM 1, 3, and 6, and their associations with the “enjoyment-of-life” score were examined using Spearman correlation.

**Results:**

We analyzed patients who underwent total (TG, n = 44), distal (DG, n = 44), proximal (PrG, n = 22), and partial gastrectomy (n = 14). Symptom burden was greatest around POD 3 across groups. TG patients showed persistently elevated scores in multiple symptoms even at POM 6. Recovery rates at POM 1, 3, and 6 were 63, 72, and 75% after TG and 79, 90, and 96% after other gastrectomies. In multivariable analysis, open TG was independently associated with failure to recover (odds ratio 0.28 [reference: robotic DG]; *p* = 0.048), whereas robotic TG was not (odds ratio 0.8; *p* = 0.742). TG and PrG patients experienced 15% BW loss through POM6; however, the enjoyment-of-life score showed no association with BW.

**Conclusions:**

Using PRO-based recovery definition, TG was associated with delayed recovery, and postoperative course varied among gastrectomy types.

**Supplementary Information:**

The online version contains supplementary material available at 10.1245/s10434-026-19733-8.

Patient-reported outcomes (PROs), which directly capture patients’ perceptions of their physical, functional, and psychological well-being, are increasingly recognized as essential tools for advancing patient-centered cancer care.^1^ Although patients frequently ask what “recovery” looks like after gastrectomy, there is limited evidence to guide preoperative counseling. Traditional metrics, such as length of hospital stay or complication rates,^2,3^ provide only indirect indicators of recovery and do not reflect patients’ actual postoperative experience. Patient-reported outcomes instead provide a direct measure of symptom burden and functional status.^4^ However, their clinical use remains limited because longitudinal PROs data after gastrectomy are scarce, and no standardized benchmarks define patient-centered recovery. Without a clear, generalizable definition, clinicians struggle to translate symptom trajectories into actionable decisions, limiting the integration of PROs into routine perioperative care.

Gastrectomy profoundly impacts patients’ quality of life for a prolonged duration. Advances in minimally invasive and robotic techniques,^5^ along with enhanced recovery pathways,^6^ are believed to facilitate early postoperative recovery.^7^ However, the duration and severity of postoperative symptom burden, as well as how these trajectories vary by surgical extent, approach, underlying diseases, and patient characteristics, remain poorly understood owing to a lack of comprehensive longitudinal PRO data.^8–10^

To address these knowledge gaps, we prospectively collected longitudinal PROs from patients undergoing gastrectomy. Through repeated assessments from the immediate postoperative period to the subacute phase, we aimed to delineate detailed symptom trajectories across different surgical procedures. By integrating these trajectories, our study sought to (1) characterize postoperative symptom burden and establish a patient-centered, generalizable definition of recovery after gastrectomy, (2) describe variations in recovery patterns by operation type and approach, and (3) identify clinical and perioperative factors associated with accelerated or delayed recovery. Ultimately, our goal is to advance a standardized framework for measuring and interpreting patient-centered recovery that can inform both clinical decision-making and future surgical quality metrics.

## Methods

### Study Design and Patient Population

This study included two independent, nonoverlapping protocols that prospectively collected PROs from patients who underwent gastrectomy at The University of Texas MD Anderson Cancer Center from October 2020 to January 2025.

The first cohort was part of the MD Anderson Symptom Inventory–Upper Gastrointestinal Surgery (MDASI-UGI-Surg) validation study,^11,12^ a prospective study designed to validate a surgery-specific symptom assessment tool. Eligible patients were ≥18 years old, able to read and write in English, and scheduled to undergo surgery for esophageal, gastric, or pancreatic disease from October 2020 to September 2024.

The second cohort consisted of patients enrolled in a concurrent quality improvement (QI) project that prospectively collected PROs from individuals undergoing gastrectomy or pancreatectomy from October 2020 to January 2025 who were not simultaneously enrolled in the validation study. This project used the MDASI–Gastrointestinal (MDASI-GI) module.^13^

For the present analysis, only patients who underwent gastrectomy were included. In both studies, PRO data were collected electronically via REDCap, a secure web-based data capture system. Surveys were administered at standardized time points: preoperatively; on postoperative days (PODs) 3, 7, 14, and 21; and at postoperative months (POMs) 1, 3, and 6. Participants received email invitations containing individualized secure links that expired after a defined period. In the MDASI-UGI-Surg validation study, dedicated research coordinators actively encouraged survey completion, whereas the QI project relied primarily on patients’ self-directed participation. Patients who did not complete at least two surveys across the perioperative period were excluded, regardless of whether a preoperative baseline assessment was available.

The Institutional Review Board of The University of Texas MD Anderson Cancer Center approved both protocols (IRB #2021-0799 and QIAB #527). Clinical data were obtained through manual review of electronic medical records, including pathological diagnosis, surgical details (operation type: total [TG], distal [DG], proximal [PrG], or partial gastrectomy [PaG]; and surgical approach: open or robotic), preoperative therapy (chemotherapy, radiation, and/or immunotherapy), postoperative complications (type and severity), length of stay, operative time, estimated blood loss, and body weight (BW). Surgical approach was defined based on the actual procedure performed; no cases of conversion from robotic to open surgery occurred during the study period.

### MDASI Instruments

Both cohorts completed MDASI-based instruments. The MDASI is a validated, patient-reported questionnaire designed to assess the severity of cancer-related symptoms and their interference with daily functioning, using a 0–10 numeric scale (0 = no symptom/interference; 10 = worst imaginable interference).

The MDASI-UGI-Surg instrument contains 28 items, comprising 13 core symptoms, nine upper gastrointestinal surgery-specific symptoms, and six interference items. The MDASI-GI instrument includes 24 items, comprising 13 identical core symptoms, five gastrointestinal-specific symptoms, and 6 identical interference items. For the unified analysis in this study, all core and interference items, as well as three overlapping module symptoms (constipation, diarrhea, and difficulty swallowing), were included, resulting in a total of 22 items analyzed (Supplementary Information 1).

### Statistical Analyses

Analyses were conducted with four main objectives. Survey response rates were calculated as the number of completed surveys divided by the number of expected surveys. First, patient enrollment, baseline characteristics, and response rates were summarized. Clinical variables were compared between open and robotic groups using the Mann-Whitney *U* test for continuous variables and Fisher exact test for categorical variables.

Second, longitudinal symptom recovery patterns after gastrectomy were described. Mean scores for each of the 22 MDASI symptom and interference items were calculated at each time point, and individual item trajectories were plotted. Because we observed substantial differences among procedure types, these results were stratified by procedure types (TG vs. other gastrectomy). To formally evaluate longitudinal differences in symptom trajectories, we performed linear mixed-effects modeling with group-by-time interactions using natural spline functions.

Third, we aimed to define “recovery” after gastrectomy using composite MDASI scores. Symptom and interference items were ranked by mean severity on POD 3 in the overall cohort, and the top five symptom items and top three interference items were used to construct composite scores for all patients. Recovery was defined as achieving both composite scores ≤3, representing resolution to none or mild symptom burden and interference. Composite score trajectories and recovery rates were compared by operation type. To identify factors associated with recovery at POM1, logistic regression analyses were performed using a prespecified parsimonious multivariable model to avoid overfitting, only using preoperative variables and operation type. Postoperative variables (operative time, estimated blood loss, complication, and length of stay) were not included to maintain temporal independence. Odds ratios and 95% CIs were estimated, adjusting for sex and a combined variable representing the operation type and surgical approach, with robotic DG as reference category (open TG, robotic TG, robotic PrG, open DG, and PaG).

Lastly, we investigated postoperative BW loss and its relationship with patients’ global quality of life. Postoperative BW changes were assessed at POM 1, 3, and 6 as a percent change from baseline, stratified by operation type. Associations between BW change and the score for “enjoyment of life,” as a representative measure of functional well-being, were examined using Spearman correlation. A two-sided *p* value < 0.05 was considered statistically significant. All analyses were conducted using R software (version 4.5.1).

## Results

A total of 147 patients were registered into the prospective collection protocol, of whom 124 provided responses at two or more assessment points, with or without a preoperative assessment, and completed the survey through POM 6. Patients with no response (n = 2) or only a single response (n = 21) were excluded, as longitudinal assessment of symptom change was not possible with fewer than two time points. Of the final cohort, 27 patients were included from the MDASI-UGI-Surg validation cohort and 97 from the QI project cohort. The overall response rate was 61.1%, with higher response rates in the MDASI-UGI-Surg validation cohort (88%) compared with the QI project cohort (53.9%; Table 1).

**Table 1:** Patient enrollment and survey response rates by study protocol

	Cases	Pre	POD3	POD7	POD14	POD21	POM1	POM3	POM6	Overall
Overall	124	79.53	59.06	76.38	51.97	59.06	67.72	48.82	46.46	61.12
UGI-Surg	27	100	92.59	100	100	96.3	100	59.26	55.56	87.96
QI project	97	74.00	50	70	39	49	59	46	44	53.88

*POD* postoperative day; *POM* postoperative month; *Pre* preoperative; *QI* quality improvement; *UGI-Surg* MD Anderson Symptom Inventory-Upper Gastrointestinal Surgery study

### Patient Characteristics

Patient characteristics are summarized in Table 2. The median age of the cohort was 64.5 years; 73 patients (58.9%) were male, and 86 (69.4%) were diagnosed with adenocarcinoma. The distribution of operation types was as follows: TG in 44 patients (35.5%), DG in 44 (35.5%), PrG in 22 (17.7%), and PaG in 14 (11.3%). A robotic approach was used in 92 patients (74.2%), but the surgical approach varied by operation type: almost all PrGs (21 robotic vs. 1 open) and PaGs (13 robotic vs. 1 open) were robotic, resulting in a significant difference between the open and robotic groups (*p* < 0.001). Postoperative complications of Clavien-Dindo grade 3 or higher occurred in ten patients (8.1%).^14^ Estimated blood loss was significantly lower in the robotic group compared with the open group (25 mL vs. 100 mL, respectively; *p* < 0.001), and the length of hospital stay was significantly shorter (3 days vs. 5 days, respectively; *p* < 0.001).

**Table 2:** Baseline characteristics of patients undergoing gastrectomy by surgical approach (open or robotic)

Variable	Overall (N = 124)	Open approach (N = 32)	Robotic approach (N = 92)	*p*
Age, years, median [IQR]	64.5 [50.8–72]	64.5 [48–70]	64.5 [52.8–73.5]	0.214
Sex (male), n (%)	73 (58.9)	18 (56.2)	55 (59.8)	0.835
BMI, kg/m^2^ median [IQR]	27.1 [24.1–31.2]	27.4 [24–31.1]	27 [24.1–31.2]	0.799
*Race, n (%)*				0.055
White	83 (66.9)	28 (87.5)	55 (59.8)	
Black	11 (8.9)	2 (6.3)	9 (9.8)	
Asian	14 (11.3)	1 (3.1)	13 (14.1)	
Pacific Islander	1 (0.8)	0 (0)	1 (1.1)	
Other	15 (12.1)	1 (3.1)	14 (15.2)	
*Ethnicity, n (%)*				0.657
Hispanic/Latino	28 (22.6)	8 (25)	20 (21.7)	
Not Hispanic/Latino	92 (74.2)	24 (75)	68 (73.9)	
Prefer not to say	4 (3.2)	0 (0)	4 (4.3)	
*Histology, n (%)*				0.078
Adenocarcinoma	86 (69.4)	28 (87.5)	58 (63)	
GIST	13 (10.5)	2 (6.3)	11 (12)	
Neuroendocrine tumor	3 (2.4)	0 (0)	3 (3.3)	
Other	22 (17.7)	2 (6.3)	20 (21.7)	
*Therapy type, n (%)*				
Neoadjuvant therapy	79 (63.7)	24 (75)	55 (59.8)	0.14
Chemotherapy	74 (59.7)	22 (68.8)	52 (56.5)	0.296
Radiation therapy	43 (34.7)	14 (43.8)	29 (31.5)	0.281
Immunotherapy	12 (9.7)	4 (12.5)	8 (8.7)	0.505
Tumor size, cm, median [IQR]	3.0 [0.9–5.4]	2.5 [0.2–4.8]	3.1 [1.8–5.4]	0.108
*Operation type, n (%)*				**<0.001**
Total gastrectomy	44 (35.5)	22 (68.8)	22 (23.9)	
Distal gastrectomy	44 (35.5)	8 (25)	36 (39.1)	
Proximal gastrectomy	22 (17.7)	1 (3.1)	21 (22.8)	
Partial gastrectomy	14 (11.3)	1 (3.1)	13 (14.1)	
Operative time, min, median [IQR]	285.5 [232.5–334.2]	269 [243–324.2]	289 [223–335]	0.851
Estimated blood loss, mL, median [IQR]	50 [25–100]	100 [50–200]	25 [20–50]	**<0.001**
*Complications, n (%)*				
None	99 (79.8)	21 (65.6)	78 (84.8)	0.068
≥ Grade III^a^	10 (8.1)	3 (9.4)	7 (7.6)	0.717
Length of hospital stay, d	4 [3–5]	5 [4–6]	3 [3, 4]	**<0.001**

Bolded *p*-values are statistically significant

^a^Clavien-Dindo classification

*BMI* body mass index; *GIST* gastrointestinal stromal tumor; *IQR* interquartile range

### Gastrectomy Symptom Burden Trends: Individual Items

**Fig. 1:**
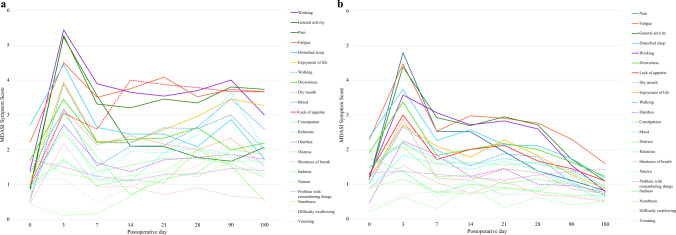
Longitudinal symptom trajectories assessed using the MD Anderson Symptom Inventory (MDASI) survey across the perioperative period. Items are ordered by their mean score on postoperative day (POD) 3, with higher scores indicating more severe symptoms. The same color indicates the same symptom across panels. Solid lines denote items included in the composite score, and dashed lines denote items not included.** a** Total gastrectomy group**. b** Nontotal gastrectomy group (distal, proximal, and partial gastrectomy groups)

Figure 1 shows longitudinal trajectories for all 22 MDASI symptom and interference items, displayed separately for TG (Fig. 1a) and non-TG procedures (Fig. 1b). Across both groups, symptom burden was high in the early postoperative phase, particularly around POD 3. The prominent symptoms in both groups at this time included pain, fatigue, dry mouth, disturbed sleep, drowsiness, and lack of appetite. In addition, substantial functional interference was observed, particularly in general activity, working, walking, and enjoyment of life.

When aggregated across all patients, the highest mean POD 3 scores were observed for pain (4.89), general activity (4.6), fatigue (4.48), working (4.04), disturbed sleep (3.91), drowsiness (3.39), lack of appetite (3.01), and enjoyment of life (3.01). The top five symptom items and top three interference items were incorporated into composite symptom and interference scores.

While symptom items commonly reported in an acute postoperative phase (first 2 weeks) were similar across the operation types, the recovery trajectory was distinct between the TG and non-TG groups. While the non-TG group demonstrated continued improvement in symptom severity toward near resolution in POM6, the TG group exhibited a slower recovery course characterized by several prolonged gastrointestinal and general symptoms (e.g., lack of appetite, dry mouth, fatigue) with functional interference (e.g., decreased general activity) remaining above the mild threshold (>3) or showing transient rebound between POM 3 and 6, representing prolonged quality of life impairment associated with the complete loss of gastric function.

### Operation Type–Specific Analyses of Recovery: Composite Scores

**Fig. 2:**
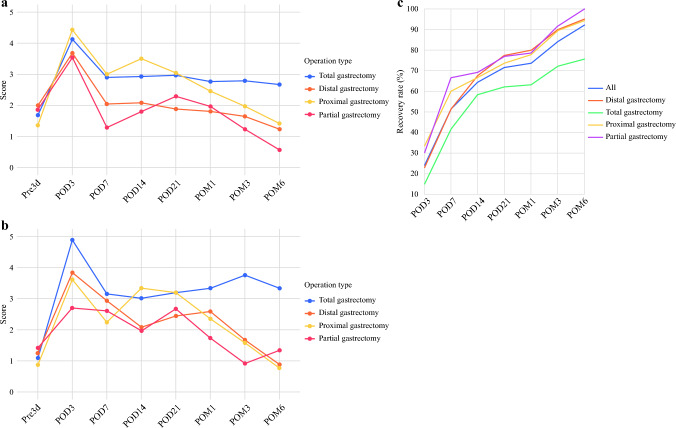
Mean composite MD Anderson Symptom Inventory (MDASI) symptom and interference trajectories by operation type (black, all types of gastrectomy; blue, total gastrectomy; orange, distal gastrectomy; yellow, proximal gastrectomy; and pink, partial gastrectomy) over time. **a** Top five symptoms (pain, fatigue, disturbed sleep, drowsiness, and lack of appetite) with the highest mean scores on postoperative day 3 (POD3), selected from core and module symptom items. **b** Top 3 interference items (general activity, working, and enjoyment of life) with the highest mean scores on POD3. **c** Trends in recovery rates from POD 3 to postoperative month 6

Mean composite symptom and interference scores by operation type are shown in Fig. 2a and b. Among operation types, the TG group demonstrated the highest mean composite scores, which remained elevated through POM 6. The DG and PaG groups showed comparable scores and similar recovery patterns over time. Notably, the PrG group exhibited scores comparable to TG during the acute-subacute postoperative phase (through approximately POM1), but their scores continued to improve thereafter, with trajectories diverging from TG by POM 3–6. Findings from linear mixed-effects modeling were consistent with these descriptive patterns, demonstrating significantly higher symptom burden after TG and PrG compared with DG during the early postoperative period (Supplementary Information 2).

Cumulative recovery rates (achieving both composite scores ≤ 3) of all patients were 74, 84, and 92%, at POM 1, 3, and 6, respectively (Fig. 2c). Recovery rates were consistently lower after TG compared with other operation types across all time points. Specifically, recovery after TG was 63, 72, and 75% at POM 1, 3, and 6, whereas recovery after non-TG procedures reached 79, 90, and 96% at the corresponding time points. These trends indicate that patients who underwent TG consistently lagged behind those who underwent other gastrectomy types in achieving postoperative recovery milestones.

In univariable analysis, open TG and postoperative complications were associated with lower odds of recovery, while Black race was associated with higher odds. The occurrence of complications of Clavien-Dindo grade 3 or higher was strongly associated with failure to achieve recovery by POM1 (Table 3). Neoadjuvant chemotherapy and tumor histology were not significantly associated with recovery in the univariable analysis. In the prespecified multivariable model, including sex and a combined variable representing operation type and surgical approach, only open TG remained significantly associated with failure to recover (odds ratio 0.28; 95% confidence interval [CI] 0.08–0.97; *p* = 0.048), whereas robotic TG was not (odds ratio 0.8; 95% CI 0.21–3.17; *p* = 0.742) compared with robotic DG as a reference.

**Table 3:** Univariable and multivariable analyses of factors associated with recovery at postoperative month 1^a^

	Univariable analysis			Multivariable analysis		
Variable	OR	95% CI	*p*	OR	95% CI	*p*
Age ≥65 years (vs. <65 years)	1.60	0.68–3.85	0.281			
Women (vs. men)	0.44	0.18–1.03	0.061	0.44	0.17–1.12	0.088
BMI ≥27 kg/m^2^ (vs. <27 kg/m^2^)	1.15	0.49–2.72	0.741			
Race (vs. White)			**0.049**			
Black	NA^b^					
Asian	2.75	0.67–18.69	0.211			
Pacific Islander	NA^b^					
Other	1.87	0.51–8.66	0.385			
Not Hispanic/Latino (vs. Hispanic/Latino)	0.81	0.27–2.19	0.690			
Histology (vs. adenocarcinoma)			0.535			
Neuroendocrine tumor	0.58	0.05–12.84	0.661			
GIST	0.65	0.19–2.63	0.512			
Other	0.45	0.15–1.39	0.154			
Neoadjuvant therapy (vs. no neoadjuvant therapy)	1.34	0.55–3.18	0.514			
Operation type and approach (vs. robotic DG)			0.307			0.371
Open TG	0.27	0.07–0.92	**0.039**	0.28	0.08–0.97	**0.048**
Robotic TG	0.81	0.22–3.15	0.750	0.80	0.21–3.17	0.742
Robotic PG	0.94	0.24–4.11	0.933	0.73	0.17–3.31	0.668
Open DG	1.62	0.22–33.14	0.680	1.41	0.18–29.41	0.769
Partial gastrectomy	0.99	0.23–5.23	0.987	1.15	0.26–6.21	0.864
Operative time ≥285 min (vs. <285 min)	0.72	0.30–1.68	0.446			
Estimated blood loss ≥50 mL (vs. <50 mL)	0.62	0.26–1.46	0.281			
Clavien–Dindo ≥ Grade III (vs. ≤ II)	0.18	0.04–0.81	**0.028**			
Length of hospital stay ≥4 days (vs. <4 days)	0.98	0.41–2.30	0.955			

Bolded *p*-values are statistically significant

^a^Logistic regression models; a higher OR indicates faster recovery

^b^Variable omitted from univariable analysis due to a small sample size

*BMI* body mass index; *DG* distal gastrectomy; *GIST* gastrointestinal stromal tumor; *OR* odds ratio; *PG* proximal gastrectomy; *TG* total gastrectomy

### Association Between BW Change and Symptom Measures

**Fig. 3:**
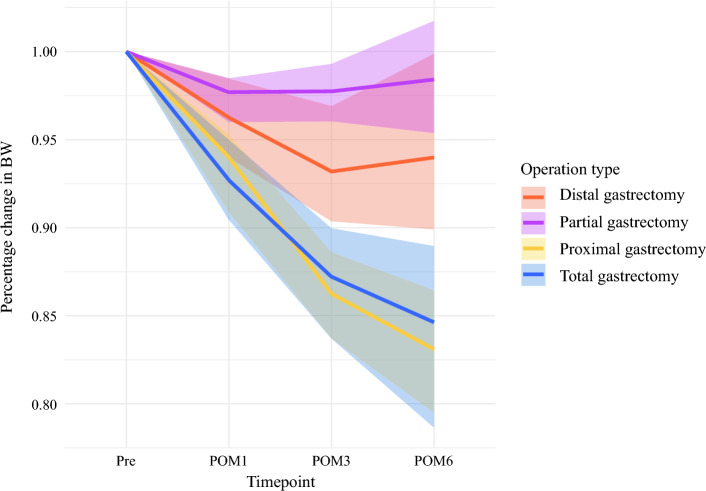
Mean percentage changes in body weight (BW) at postoperative months (POMs) 1, 3, and 6 compared with preoperative baseline (Pre)

Figure 3 illustrates postoperative BW trajectories by operation type. BW declined across all groups, with both the TG and PrG groups exhibiting a progressive decrease through POM 6, reaching approximately 15% loss from baseline. In contrast, BW loss plateaued earlier after DG and PaG, stabilizing by POM3 with less than 10% total loss. When Spearman’s analyses were applied, no significant association was observed between BW loss and the “enjoyment of life” item score at any time point (all |ρ| < 0.3, *p* > 0.05), suggesting that although BW loss is common and often substantial after gastrectomy, it did not correlate with patient-reported global quality of life.

## Discussion

This study is the first to longitudinally characterize postoperative recovery after gastrectomy using the MDASI framework and to establish a composite, PRO-based recovery definition. By capturing eight time points, our analysis provides a granular view of early and subacute recovery dynamics that cannot be appreciated through single-time-point or late-phase assessments reported previously.^15–19^ Importantly, the proposed definition of recovery based on PRO data in this study operationalized PRO data into a quantifiable recovery index, transforming symptom trajectories into clinically interpretable information that bridges subjective patient experience with objective surgical outcomes. Most notably, while non-TG groups reached cumulative recovery rates exceeding 90% by POM 3, the TG group showed a slower trajectory, with recovery reaching only 75% by POM 6, representing distinct recovery patterns after TG compared with other less extensive gastrectomy types. Robotic approaches may mitigate acute and subacute symptoms after TG.

The strengths of this study lie in its comprehensive design and analytic depth. We demonstrated distinct symptom-recovery profiles by surgical procedure and introduced a standardized, multidimensional PRO-based recovery framework capable of capturing the complexity of postoperative recovery. Although prior studies have used PRO measures in evaluating postoperative recovery,^20^ few have provided a recovery definition that comprehensively incorporates multiple symptom domains or is directly applicable to gastrectomy.^21^ The observed multiphase recovery pattern underscores the limitations of single-time-point assessments and highlights the importance of continuous symptom monitoring.^22^ In addition, conducting this study at a single high-volume cancer center with standardized perioperative management,^6^ and a prospective, protocolized PRO-collection schedule minimized confounding from variability in postoperative care and allowed for clearer comparisons across surgical procedures. As an initial step toward defining PRO-based recovery trajectories after gastrectomy, this study of high-frequency, longitudinal PROs provides valuable benchmark data for future clinical applications and research development.

Our key finding is that TG remains associated with more prolonged and variable symptom recovery compared with other gastrectomy procedures, even in the current era of enhanced recovery pathways.^23^ This finding aligns with prior evidence showing prolonged effects of impaired quality of life after TG.^24–28^ In addition, the absence of a gastric reservoir results in rapid transit of ingested food into the small intestine, predisposing patients to early and late dumping symptoms, such as palpitations, dizziness, and postprandial fatigue, which further impair postoperative recovery.^29^ Although most DGs performed in our US center require subtotal gastrectomy by removing at least 70% of gastric volume,^30^ we observed very different recovery trajectories between TG and DG in this study, proving the benefits of preservation of gastric function even with the small volume. BW loss after DG was modest (approximately 7%) and plateaued at POM 3. Indeed, patients’ recovery after DG was similar to recovery after partial wedge gastrectomy. The message here is clear—the proximal portion of the stomach with intact gastroesophageal junction is important.

In contrast, it remains controversial whether PrG, as a function-preserving gastrectomy, provides better quality of life compared with TG. In PrG, the preserved distal stomach may serve as a food reservoir and produce acid, intrinsic factor, and gastric hormones, such as ghrelin, potentially mitigating postoperative symptoms.^31^ In addition, double-tract reconstruction has been increasingly adopted in recent years as a strategy to reduce reflux after PrG. This shift in reconstruction technique may partly explain differences between earlier reports and more recent findings.^15^ The KLASS-05 randomized controlled trial comparing laparoscopic TG and PrG in 138 patients with cT1N0 proximal gastric cancer demonstrated reduced vitamin B12 supplementation after PrG but similar hemoglobin levels and overall survival, with only limited improvements in quality of life measures.^32,33^ Additionally, a recent Korean cross-sectional PRO survey study reported notably poor quality of life after PrG due to increased upper gastrointestinal symptoms, including reflux.^34^

In this present study, PrG was associated with a continuous decline in BW similar to TG, averaging more than 15% loss through 6 months. Neither TG nor PrG appeared to reach a clear recovery plateau by the 6-month time point, suggesting that longer follow-up is required to fully characterize the timing and extent of recovery after these procedures. However, composite PRO-based recovery trajectories indicated that patients after PrG began to show improvement by POM 3 and achieved scores comparable to DG at POM 6. Interestingly, in our ongoing multi-institutional prospective PRO study comparing minimally invasive (mostly robotic) PrG and TG,^35^ quality of life outcomes were comparable at 3 months.^36^ This difference from the present findings may be explained by variation in surgical approach, as PrG in the current cohort was almost exclusively robotic, whereas TG included a substantial proportion of open procedures, limiting the validity of direct comparisons. This discrepancy between BW loss and PRO outcomes was supported by our finding that BW loss did not correlate with “enjoyment of life” scores, suggesting that symptom-based recovery and nutritional recovery may reflect distinct aspects of postoperative recovery. The BW–QoL analysis was exploratory and focused on “enjoyment of life” as a representative global measure. No consistent associations were observed across other items, suggesting that BW loss may not directly reflect patient-perceived recovery. Given the relatively small sample size of the PrG subgroup, the present analysis is underpowered to draw definitive conclusions regarding PrG-specific recovery patterns, and these findings should be considered exploratory. These findings underscore the need for further prospective studies to clarify the patient-centered benefits of PrG and minimally invasive techniques.

This study has several limitations. First, the possibility of selection bias cannot be excluded as participation required survey completion at multiple time points, which may have favored patients with smoother postoperative courses or stronger engagement in follow-up, and exclusion of patients with insufficient longitudinal PRO data may have further introduced selection bias. In addition, the merged dataset included two cohorts with different response rates, which may introduce non-response bias and heterogeneity that were not formally adjusted for. Second, this study was conducted at a single institution, which may limit the generalizability of the findings. However, this setting also has advantages: procedures were performed at a high-volume center by a limited number of surgeons, allowing for a more accurate assessment of the pure impact of the surgical approach. Third, although the longitudinal design captured detailed perioperative dynamics, follow-up was limited to 6 months, and longer-term functional adaptation or late symptom changes could not be assessed. In particular, differences in surgical approach across procedure types may introduce residual confounding, and the observed associations should be interpreted with caution. In addition, the current analysis cannot fully disentangle the relative contributions of surgical approach and postoperative morbidity to recovery outcomes. While the multivariable model was intentionally parsimonious to avoid overfitting, this design limited the inclusion of additional clinical covariates that may also influence recovery. Fourth, the recovery threshold used in this study was empirically derived within this dataset and has not been externally validated or anchored to patient-centered benchmarks, and therefore its clinical interpretability remains limited. Finally, PRO data inherently depend on patient self-reporting and response compliance, introducing potential recall or reporting biases despite the standardized MDASI structure. Nonetheless, these limitations are balanced by the study’s strength in high-frequency, standardized PRO collection, providing a strong foundation for future multicenter validation and extended longitudinal studies to establish the broader utility of this MDASI-based recovery measure.

## Conclusions

This study comprehensively evaluated postoperative symptom trajectories following gastrectomy using the MDASI instruments and defined postoperative recovery using a composite score. We found that symptom trajectories differed by operation type, with TG associated with the slowest recovery. BW changes followed a pattern distinct from MDASI symptom trajectories, highlighting that BW loss alone does not fully capture patient-perceived recovery. These findings provide a foundation for tailoring postoperative care to individual patient needs, supporting more personalized interventions after gastrectomy.

## Supplementary Information

Below is the link to the electronic supplementary material.

Supplementary file1 (DOCX 18 KB)
