# Supplementary material for: Defining Recovery After Gastrectomy: Insights from Longitudinally Collected Patient-Reported Outcomes
Source: Ann Surg Oncol. 2026 Apr 30;33(8):7302–11. doi: 10.1245/s10434-026-19733-8 (PMC13337917; doi:10.1245/s10434-026-19733-8)
Supplement: Supplementary file 1 — Supplementary file1 (DOCX 18 KB) [file 10434_2026_19733_MOESM1_ESM.docx]

**Supplementary Information 1** MDASI instruments

Symptoms and interference are rated for the last 24 h on 0 (symptom does not present or no interference) to 10 scales (symptom as bad as you can imagine or complete interference).

The core and module symptoms are assessed by the question 'How severe are your symptoms?', while the interference items are evaluated using 'How have your symptoms interfered with your life?'

| Core Symptoms |
| --- |
| 1. Pain |
| 1. Fatigue |
| 1. Nausea |
| 1. Disturbed sleep |
| 1. Distress |
| 1. Shortness of breath |
| 1. Problem with remembering things |
| 1. Lack of appetite |
| 1. Drowsiness |
| 1. Dry mouth |
| 1. Sadness |
| 1. Vomiting |
| 1. Numbness or tingling |
| Module Symptoms |
| 1. Difficulty swallowing |
| 1. Diarrhea |
| 1. Constipation |
| Interference Items |
| 1. General activity |
| 1. Mood |
| 1. Working (including work around the house) |
| 1. Relations with other people |
| 1. Walking |
| 1. Enjoyment of life |

**Supplementary Information 2** Linear mixed-effects model

Longitudinal changes in symptom scores were analyzed using linear mixed-effects models with natural cubic splines (degrees of freedom = 4, with 3 knots) to flexibly model nonlinear time trends. The spline degree was selected based on model fit criteria. Random intercepts for each patient were included to account for within-subject correlations. Fixed effects included time splines, operation type, approach, sex, histology, and complications. Missing data were excluded from analyses without imputation.

These models confirmed that TG (estimate = 2.19, p = 0.02) and PrG (estimate = 2.59, p = 0.02) had significantly higher symptom scores compared with DG during the mid postoperative period (POD 18–44). Similarly, TG showed significantly higher interference scores during both the mid (estimate = 2.45, p = 0.04) and later postoperative periods (POD 44–180; estimate = 2.09, p = 0.003).
